# Supplementary material for: Effects of Reduced Phosphate Fertilizer and Increased Trichoderma Application on the Growth, Yield, and Quality of Pepper
Source: Plants (Basel). 2023 Aug 19;12(16):2998. doi: 10.3390/plants12162998 (PMC10460083; doi:10.3390/plants12162998)
Supplement: Supplementary file 1 [file plants-12-02998-s001.zip › plants-2435902-supplementary.pdf]

Table S1 Effects of different levels of phosphate, *Trichoderma*, and their interaction on plant growth

Green fruit stage	Different levels of phosphate		*Trichoderma*		Different levels of phosphate * *Trichoderma*		Red fruit stage	Different levels of phosphate		*Trichoderma*		Different levels of phosphate * *Trichoderma*	
	F	*P*	*F*	*P*	*F*	*P*		*F*	*P*	*F*	*P*	*F*	*P*
Plant height	3.498	**0.04**	6.069	**0.025**	1.053	0.396	Plant height	1.246	0.326	8.103	0.012	0.13	0.941
Stem diameter	1.919	0.167	13.431	**0.002**	0.716	0.557	Stem diameter	1.514	0.249	3.535	0.078	0.572	0.641
SPAD	5.418	**0.009**	31.611	**0.000**	0.473	0.705	SPAD	3.108	0.056	0.871	0.364	0.554	0.653
Dry weight of roots	2.564	**0.091**	2.666	**0.122**	1.602	0.228	Dry weight of roots	12.251	**0.000**	18.846	**0.001**	0.641	0.600
Dry weight of stems	4.747	**0.015**	4.543	**0.049**	0.586	0.633	Dry weight of stems	44.453	**0.000**	693.338	**0.000**	48.437	**0.000**
Dry weight of leave	1.449	0.266	8.348	**0.011**	2.121	0.138	Dry weight of leave	38.009	**0.000**	432.496	**0.000**	11.671	**0.000**
Dry weight of fruits	1.464	0.262	30.620	**0.000**	6.108	**0.006**	Dry weight of fruits	8.378	**0.001**	104.524	**0.000**	0.687	0.573

Note: *p<0.05; **p<0.01; ***p<0.001, For each parameter, p<0.05 is in bold.

Table S2 Effects of different levels of phosphate, *Trichoderma*, and their interaction on plant nutrition

Green fruit stage	Different levels of phosphate		*Trichoderma*		Different levels of phosphate * *Trichoderma*		Red fruit stage	Different levels of phosphate		*Trichoderma*		Different levels of phosphate * *Trichoderma*	
	F	*P*	*F*	*P*	*F*	*P*		*F*	*P*	*F*	*P*	*F*	*P*
The N content of whole plant	3.871	**0.03**	42.289	**0.000**	12.153	**0.000**	The N content of whole plant	23.484	**0.000**	159.153	**0.000**	24.469	**0.000**
The P content of whole plant	4.317	**0.021**	47.057	**0.000**	8.445	**0.001**	The P content of whole plant	46.159	**0.000**	437.041	**0.000**	11.033	**0.000**
The K content of whole plant	3.446	**0.042**	42.349	**0.000**	9.24	**0.001**	The K content of whole plant	34.722	**0.000**	297.861	**0.000**	3.448	**0.042**

Note: *p<0.05; **p<0.01; ***p<0.001, For each parameter, p<0.05 is in bold.

Table S3 Effects of different levels of phosphate, *Trichoderma*, and their interaction on pepper fruit quality

Green fruit stage	Different levels of phosphate		*Trichoderma*		Different levels of phosphate * *Trichoderma*		Red fruit stage	Different levels of phosphate		*Trichoderma*		Different levels of phosphate * *Trichoderma*	
	F	*P*	*F*	*P*	*F*	*P*		*F*	*P*	*F*	*P*	*F*	*P*
Soluble protein	0.830	0.497	14.724	**0.001**	1.880	0.174	Soluble protein	14.759	**0.000**	7.757	**0.013**	0.376	0.772
Vitamin C	373.672	**0.000**	23.434	**0.000**	469.458	**0.000**	Vitamin C	39.240	**0.000**	48.440	**0.000**	8.191	**0.002**
Soluble sugar	3.298	**0.048**	2.263	0.152	2.891	0.068	Soluble sugar	8.988	**0.001**	19.439	**0.000**	0.710	0.560
Capsaicin	78.884	**0.000**	295.505	0.000	76.739	**0.000**	Capsaicin	21.715	**0.000**	61.634	**0.000**	24.916	**0.000**
Capsanthin	18.345	**0.000**	11.929	**0.003**	5.270	**0.010**	Capsanthin	63.057	**0.000**	51.586	**0.000**	26.744	**0.000**

Note: *p<0.05; **p<0.01; ***p<0.001, For each parameter, p<0.05 is in bold.

Table S4 Effects of different levels of phosphate, *Trichoderma*, and their interaction on soil fertility

Green fruit stage	Different levels of phosphate		*Trichoderma*		Different levels of phosphate * *Trichoderma*		Red fruit stage	Different levels of phosphate		*Trichoderma*		Different levels of phosphate * *Trichoderma*	
	F	*P*	*F*	*P*	*F*	*P*		*F*	*P*	*F*	*P*	*F*	*P*
Total N content	2.323	0.114	0.001	0.980	9.439	**0.001**	Total N content	4.568	**0.017**	2.291	0.150	6.815	**0.004**
Alkali hydrolyzed N content	1.977	0.158	0.882	0.362	6.562	**0.004**	Alkali hydrolyzed N content	3.680	**0.034**	11.757	0.204	0.440	0.728
Total P content	12.800	**0.000**	1.285	0.274	15.116	0.000	Total P content	48.206	**0.000**	9.540	**0.007**	76.023	**0.000**
Available P content	7.592	**0.002**	4.239	0.056	1.214	0.337	Available P content	10.055	**0.001**	8.892	**0.009**	21.139	**0.000**
Total K content	15.454	**0.000**	8.696	**0.009**	1.817	0.185	Total K content	0.293	0.830	2.605	0.126	3.519	**0.039**
Available K content	14.035	**0.000**	182.911	**0.000**	23.140	**0.000**	Available K content	6.274	**0.005**	11.588	0.004	3.925	**0.028**

Note: *$p<0.05$; **$p<0.01$; ***$p<0.001$, For each parameter, $p<0.05$ is in bold.
